# Disruption of the thrombospondin-2 gene alters the lamellar morphology but does not permit vascularization of the adult mouse lumbar disc

**DOI:** 10.1186/ar2483

**Published:** 2008-08-21

**Authors:** Helen E Gruber, Paul Bornstein, E Helene Sage, Jane A Ingram, Natalia Zinchenko, H James Norton, Edward N Hanley

**Affiliations:** 1 Department of Orthopaedic Surgery, Carolinas Medical Center, PO Box 32861, Charlotte, NC 28232, USA; 2 Departments of Medicine and Biochemistry, University of Washington, Seattle, WA 98195, USA; 3 Hope Heart Program, The Benaroya Institute at Virginia Mason, 1201 Ninth Avenue, Seattle, WA 98101-2795, USA; 4 Department of Biostatistics, Carolinas Medical Center, PO Box 332861, Charlotte, NC 28232, USA

## Abstract

**Introduction:**

The biological basis for the avascular state of the intervertebral disc is not well understood. Previous work has suggested that the presence of thrombospondin-1 (TSP-1), a matricellular protein, in the outer annulus reflects a role for this protein in conferring an avascular status to the disc. In the present study we have examined thrombospondin-2 (TSP-2), a matricellular protein with recognized anti-angiogenic activity *in vivo *and *in vitro*.

**Methods:**

We examined both the location and expression of TSP-2 in the human disc, and its location in the disc and bordering soft tissues of 5-month-old normal wild-type (WT) mice and of mice with a targeted disruption of the TSP-2 gene. Immunohistochemistry and quantitative histology were utilized in this study.

**Results:**

TSP-2 was found to be present in some, but not all, annulus cells of the human annulus and the mouse annulus. Although there was no difference in the number of disc cells in the annulus of TSP-2-null mice compared with that of WT animals, polarized light microscopy revealed a more irregular lamellar collagen structure in null mouse discs compared with WT mouse discs. Additionally, vascular beds at the margins of discs of TSP-2-null mice were substantially more irregular than those of WT animals. Counts of platelet endothelial cell adhesion molecule-1-positive blood vessels in the tissue margin bordering the ventral annulus showed a significantly larger vascular bed in the tissue bordering the disc of TSP-2-null mice compared with that of WT mice (*P *= 0.0002). There was, however, no vascular ingrowth into discs of the TSP-2-null mice.

**Conclusion:**

These data confirm a role for TSP-2 in the morphology of the disc and suggest the presence of other inhibitors of angiogenesis in the disc. We have shown that although an increase in vasculature was present in the TSP-2-null tissue in the margin of the disc, vascular ingrowth into the body of the disc did not occur. Our results point to the need for future research to understand the transition from the well-vascularized status of the fetal and young discs to the avascular state of the adult human disc or the small mammalian disc.

## Introduction

The thrombospondins (TSPs) are multifunctional matricellular proteins; TSP-1 and TSP-2 have strong anti-angiogenic properties, are present in a number of tissues where they bind to the extracellular matrix (ECM) and, in turn, are themselves able to bind receptors, enzymes, cytokines, proteases, and other ECM proteins [1-6]. TSP-1 and TSP-2 bind matrix metalloproteinase-2, and thereby act to clear this matrix metalloproteinase from the pericellular ECM [5]. Both TSP-1 and TSP-2 function in the cellular response to injury, but only TSP-1 is capable of activating the small latent transforming growth factor beta complex [7,8].

Previous studies have shown that mice with a disruption of the TSP-2 gene exhibit disordered collagen fibrillogenesis, fragile skin, ligament and tendon laxity, and increased vascularity [4]. Recent work has also shown that the TSP-2-null mouse has a reduction in tissue transglutaminase, an enzyme that acts to introduce covalent intermolecular cross-links in collagen and other proteins; this finding accounts in part for the matrix abnormalities seen in the TSP-2-null mouse, such as fragile skin and lax ligaments [2]. TSP-2-null mice also exhibit significantly greater vascularity in adult and embryonic adipose tissue, and in adult and neonatal dermis [4].

Bone studies have shown that TSP-2-null mice have increased cortical density in long bones, and a mid-diaphyseal endosteal bone formation rate that is increased compared with that of wild-type (WT) mice [9]. TSP-2-null mice also exhibit an elevated bone formation rate (compared with that of WT mice) following mechanical loading [10].

TSP-1 is present in the outer annulus of both human and sand rat discs and, at apparently lower levels, in the inner annulus [11]. This work is suggestive of a role for TSP-1 in the avascular status of the disc [11].

The biological basis for the avascular state of the human adult disc is not well understood, but this question is important because the resulting lowered nutritive state of the disc might be a factor in disc degeneration [12]. Nutrients are believed to reach cells within the disc predominantly through the vertebral endplate, and disc cells are kept viable by nutrients moving by diffusion through the disc matrix.

Several recent studies have utilized murine cervical, lumbar or tail discs as experimental models. The reader is referred to recent reports that provide useful histologic data [13-16] or biomechanical data [17] on the age-related changes in the normal mouse disc.

The objective of the present work was to examine mice with a targeted disruption of the TSP-2 gene to determine whether mice lacking TSP-2 would show enhanced vascularity of the adult annulus. We first determined the immunolocalization of TSP-2 in the human disc and the normal mouse disc, and subsequently examined mice with a targeted disruption of the TSP-2 gene with respect to the morphology and cellularity of the annulus, the presence of vascular beds within the disc, and vascularity of the soft tissue margin of the disc that serves to supply nutrition to the disc via diffusion. Our studies confirm the expression of TSP-2 in both the human annulus and the WT mouse annulus, but show no vascular ingrowth into the discs of TSP-2-null mice.

## Materials and methods

### Clinical study population

The experimental study of disc specimens was approved prospectively by the authors' Human Subjects Institutional Review Board at Carolinas Medical Center. The need for informed consent was waived since disc tissue was removed as part of routine surgical practice. The Thompson grading system is used to score disc degeneration over the spectrum of stages from a healthy disc (Thompson grade I) to discs with advanced degeneration (Thompson grade V) [18].

Patient specimens were derived from surgical disc procedures performed on individuals with herniated discs and degenerative disc disease. Surgical specimens were transported to the laboratory in sterile tissue culture medium, less than 30 minutes after surgical removal, and were placed in 10% neutral buffered formalin for no longer than 24 hours. Care was taken to remove all granulation tissue and to sample only disc tissue. Nonsurgical control donor disc specimens were obtained via the National Cancer Institute Cooperative Human Tissue Network; the specimens were shipped overnight to the laboratory in sterile tissue culture medium and were processed as described below. Specimen procurement from the Cooperative Human Tissue Network was included in our approved protocol by our human subjects Institutional Review board.

Human disc tissues were processed undecalcified and embedded in paraffin, and were processed for immunohistochemistry as described below.

### TSP-2 immunolocalization in human disc specimens

Four specimens of human disc tissue were utilized for localization of TSP-2 with immunocytochemistry: a surgical specimen from a 16-year-old female, L3 to L4 (Thompson grade II); a surgical specimen from a 42-year-old female, C5 to C6 (Thompson grade III); a Cooperative Human Tissue Network specimen from a 40-year-old male (Thompson grade II); and a Cooperative Human Tissue Network specimen from L5 to S1 from a 33-year-old female, L3 to L4 (Thompson grade IV).

### Gene expression studies in human disc cells

Human disc tissue, annulus cells in monolayer, and annulus cells in three-dimensional culture were assayed for gene expression using the Affymetrix microarray system (Affymetrix, Santa Clara, CA 95051, USA). Cells from the annulus were examined from four subjects: a 52-year-old female, Thompson grade III; a 63-year-old male, Thompson grade IV; a 65-year-old female, Thompson grade IV; and a 33-year-old female control donor, Thompson grade III.

Disc tissue was studied using laser capture microdissection to harvest cells, followed by microarray analysis as previously described [19]. Cultured cells were placed in Extraction Buffer from the PicoPure RNA Isolation Kit (Arcturus, Mountainview, CA, USA). Total RNA was extracted from the tissue according to instructions in the PicoPure RNA Isolation Kit, reverse-transcribed to double-stranded cDNA, subjected to two rounds of transcription, and hybridized to the DNA microarray in the Affymetrix Fluidics Station 400. Affymetrix human U133 X3P arrays were used. The GCOS Affymetrix GeneChip Operating System (version 1.2) was used to determine gene expression levels for TSP-2 (NM_003247.1).

### Animal studies

Animal studies were performed after approval by the Institutional Animal Care and Use Committee at the University of Washington. The WT control mice and the TSP-2-null mice studied here have been described previously [4].

### Light microscopy studies of disc tissues

Light microscopy was performed on the lumbar and thoracic spines of four WT mice and four TSP-2-null mice, aged 5 months. Specimens were fixed in either 10% neutral buffered formalin or 70% ethanol, and were decalcified in a solution of 22.5% formic acid (Allegiance, McGraw Park, IL, USA) and 10% sodium citrate (Sigma, St Louis, MO, USA). Complete decalcification was determined by radiography. The spine was cut in sagittal section, embedded in paraffin, and sectioned at 4 μm. Sections were stained with Masson-trichrome stain for the evaluation of general disc features, for polarized light microscopy, and for cell counts.

### TSP-1 and TSP-2 in the mouse disc

TSP-1 was identified in the mouse disc with an antibody that recognizes primarily TSP-1 (AB-4, Clone A6.1; LabVision Corporation, Freemont, CA, USA) at a concentration of 8 μg/ml, as previously described [11]. The negative control used with each experiment was mouse IgG_1 _(Dako-Cytomation, Carpinteria, CA, USA) used at the same concentration. Positive controls (skin and breast tissue) were also included.

Immunolocalization of TSP-2 was performed with an anti-TSP-2 antibody specific for TSP-2, as previously described [20], with antigen retrieval. Antigen retrieval was performed using Dako Target Retrieval Solution, pH 6.0, for 20 minutes at 95°C, followed by cooling for 20 minutes. Negative controls were processed in the absence of the primary antibody.

### Scoring of PECAM-1-positive blood vessels

Platelet endothelial cell adhesion molecule-1 (PECAM-1) was identified as follows: sections were deparaffinized in xylene (Allegiance) and were rehydrated through graded concentrations of alcohol (AAPER, Shelbyville, KY, USA) to distilled water. As shown previously, antigen retrieval methods were required [21]; antigen retrieval was performed using Dako Target Retrieval Solution, pH 6.0, for 20 minutes at 95°C, followed by cooling for 20 minutes. Endogenous peroxidase was blocked with 3% H_2_O_2 _(Sigma). Slides were incubated overnight at 4°C with anti-PECAM-1 IgG (Santa Cruz Biotechnology, Santa Cruz, CA, USA) at a 1:100 dilution. Goat IgG (Vector Laboratories, Burlingame, CA, USA) was used as a negative control. The secondary antibody was biotinylated rabbit anti-goat IgG (Vector) applied for 20 minutes, followed by peroxidase-conjugated streptavidin (Dako) for 10 min and Vector NovaRed (Vector) for 5 minutes. Slides were rinsed in water, counterstained with light green (Polysciences, Warrington, PA, USA), dehydrated, cleared, and mounted with resinous mounting media. Control tissues included mouse spleen and human tonsil.

The number of PECAM-1-positive vascular structures in 40× magnification fields was scored along the border of the ventral disc margin. Only tissue bordering the disc (not including adjacent end plates) was scored. The margins of 20 discs for WT mice and of 15 discs for TSP-2-null mice were evaluated.

### Quantitative histomorphometry

Histomorphometry was performed on the annulus of lumbar discs to determine cell densities in WT mice and TSP-null mice. Quantitative histomorphometry was performed on tissue sections stained with Masson-trichrome dye using the OsteoMeasure system (OsteoMetrics, Atlanta, GA, USA).

### Statistical analyses

Standard statistical methods employed SAS software (version 9.1; SAS Institute, Cary, NC, USA), and GraphPad InStat^® ^(version 3.06; GraphPad Inc., San Diego, CA, USA). *P *< 0.05 was considered statistically significant. Analyses performed included calculation of descriptive statistics and the Wilcoxon rank sum test, which was utilized to assess data from counts of PECAM-positive blood vessels in the soft tissue of the disc margins. Data are expressed as the mean ± standard error of the mean (number).

## Results

### TSP-2 and its gene expression in human discs

**Figure 1:**
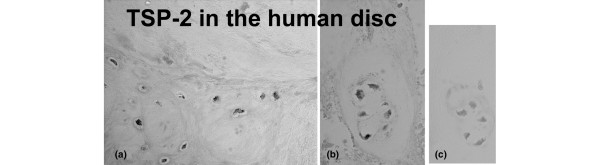
Location of thrombospondin-2 in the human annulus. **(a) **Positive identification of thrombospondin-2 (TSP-2) is present in some cells in the outer annulus. **(b) **Localization was also positive for cells in the inner annulus, including cells present in clusters. **(c) **Adjacent section processed in the absence of antibody as a negative control. Magnification, ×300.

Previous studies of TSP in the human disc and the sand rat disc – with an antibody that recognized primarily TSP-1 – showed TSP-1 in the outer annulus, and some cells with reactivity in the inner annulus [11]. In the present study, TSP-2 was identified in some, but not all, outer and inner annulus cells in the human disc (Figures 1a,b; Figure 1c presents a negative control). Affymetrix gene array expression analysis also provided independent confirmation of the expression of TSP-2 in the annulus of four human specimens (Thompson grades III and IV). The mean relative gene expression level was 8,539 ± 2,617 (n = 4).

### TSP in the mouse lumbar disc

**Figure 2:**
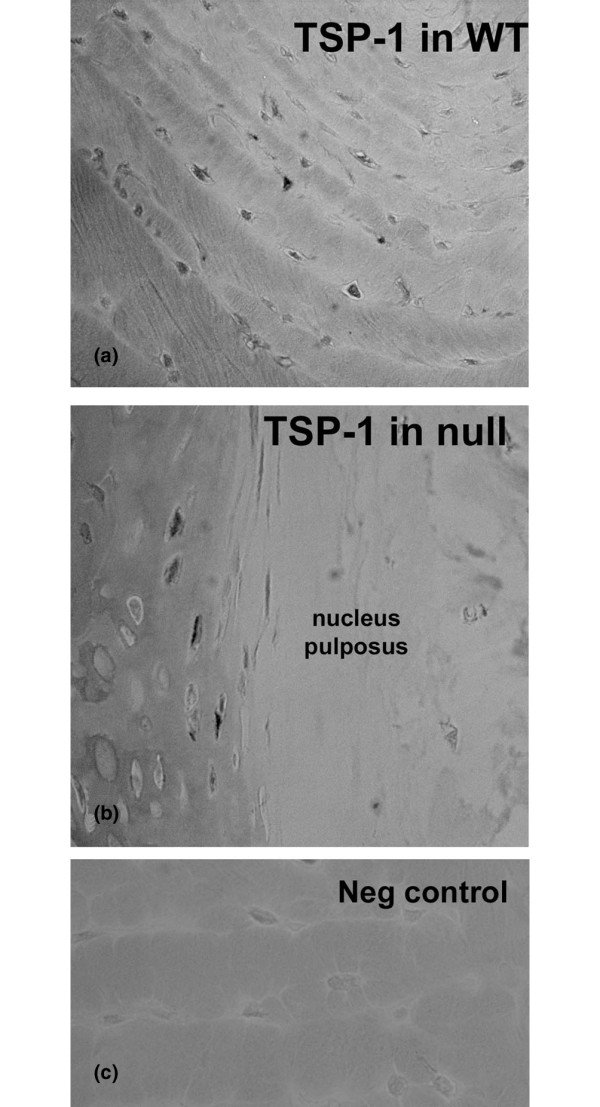
Location of thrombospondin-1 in the annulus of the mouse intervertebral disc. Dark-stained cells show localization of thrombospondin-1 (TSP-1) in **(a) **the outer annulus of wild-type (WT) mice and **(b) **the annulus adjacent to the central portion of the nucleus pulposus. **(c) **Negative control. Magnification, ×300.

In the mouse disc, TSP-1 was located in some, but not all, cells in annulus tissue from both WT mice (Figure 2a) and TSP-2-null mice (Figure 2b). Figure 2c presents a negative control.

**Figure 3:**
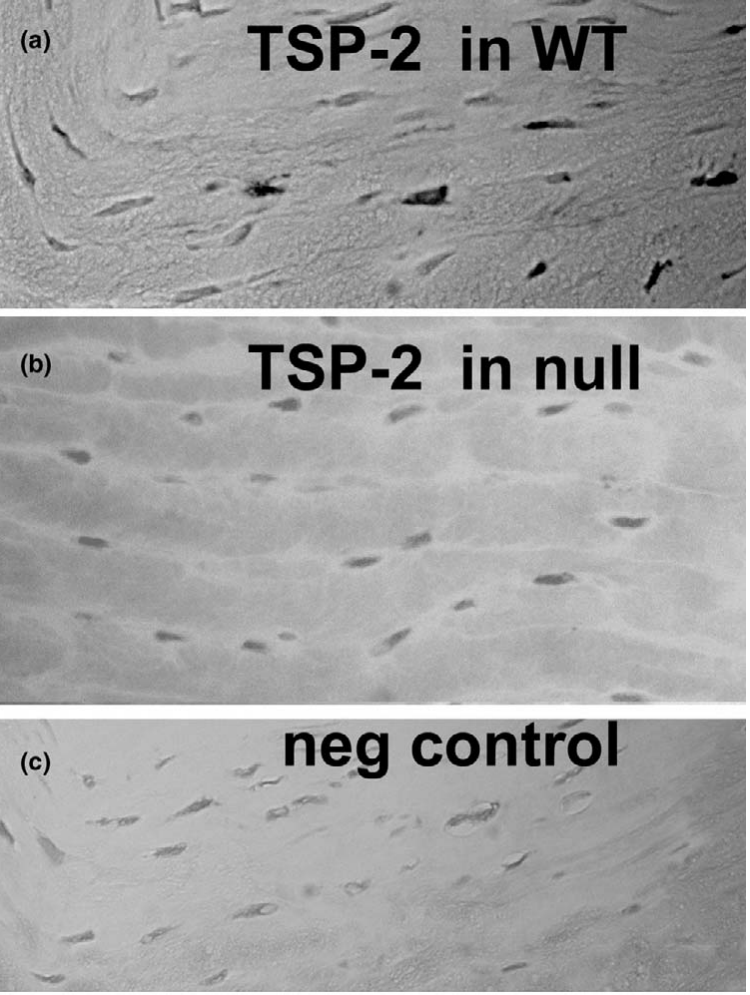
Location of thrombospondin-2 in the mouse annulus. **(a) **Thrombospondin-2 (TSP-2) is present in many cells in the annulus of wild-type (WT) mice. **(b) **TSP-2 is absent in cells of the annulus in TSP-2-null mice. **(c) **Negative control processed in the absence of primary antibody. Magnification, ×300.

TSP-2 was present in some cells in the outer annulus of the disc in WT mice (Figure 3a) and in some cells in the region between the central part of the nucleus pulposus and the adjacent vertebral endplate (similar to the localization pattern shown in Figure 2b for TSP-1) (data not shown). As expected, TSP-2 was not observed in annulus specimens from TSP-2-null mice (Figure 3b). A negative control section is shown in Figure 3c.

### Annulus cell numbers in lumbar discs of WT mice and TSP-2-null mice

**Figure 4:**
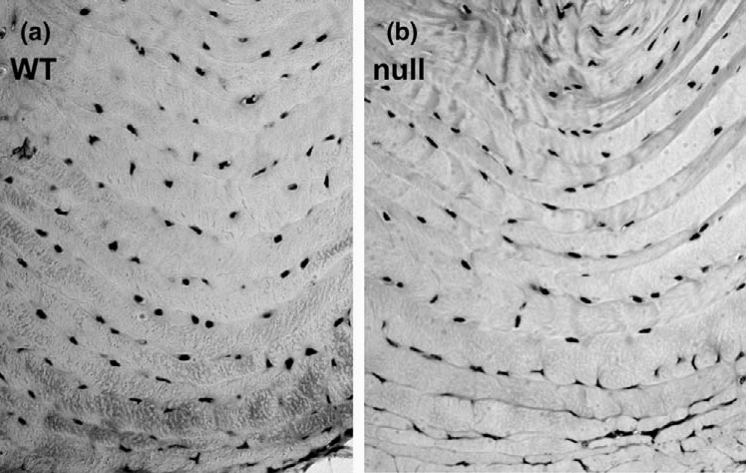
Morphologic features of wild-type and thrombospondin-2-null mouse ventral annuli. The morphologic features of the **(a) **wild-type (WT) mouse ventral annuli and **(b) **thrombospondin-2-null mouse ventral annuli are similar. Masson-trichrome stain, magnification, ×300.

Seven lumbar discs were examined from levels L1 to the L6-sacrum for each spine from each mouse. Routine staining with Masson-trichrome dye showed that neither WT mice nor TSP-2-null discs exhibited vascularity in dorsal or ventral portions of the annulus. As shown in Figure 4, the cellular density also appeared to be similar. This finding was confirmed by quantitative histomorphometric cell counts, which showed no differences in ventral annulus cell densities (WT mice, 2,990 cells/mm^2 ^± 353 (n = 4) versus TSP-2-null mice, 2,708 ± 192 (n = 4)).

### Morphology of collagen in discs of WT mice and TSP-2-null mice

**Figure 5:**
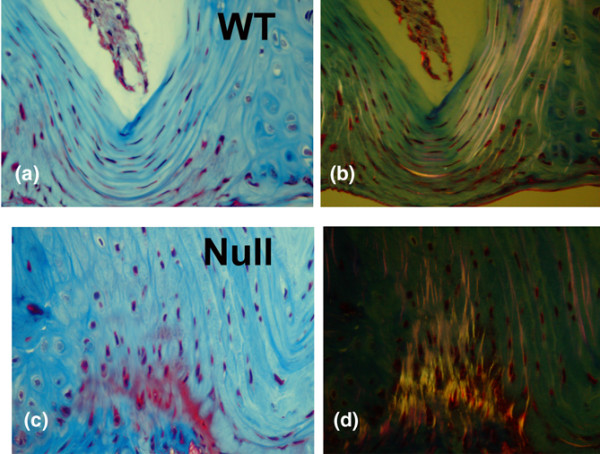
Morphology of collagen lamellae in the annulus of a mouse discs. The morphology was examined with Masson-trichrome stain and with polarized light microscopy of the same field. **(a) **and **(b) **Annulus of the wild-type (WT) mouse shows regular, even lamellar layers. **(c) **and **(d) **Annulus of the null mouse shows less regular polarized light patterns in (d). Thoracic disc, magnification, ×300.

Examination of collagen in the lamellar regions of the discs of WT mice and TSP-2-null mice was performed using Masson-trichrome-stained sections viewed with regular light microscopy and with polarizing light microscopy. This technique showed a regular, even pattern of collagens in the annuli of discs from WT mice (Figure 5a,b). In contrast, discs from TSP-2-null mice were characterized by a more irregular (woven) birefringent pattern compared with that of discs from WT mice (Figure 5c,d).

### Peripheral vascularity around lumbar discs of WT mice and TSP-2-null mice

**Figure 6:**
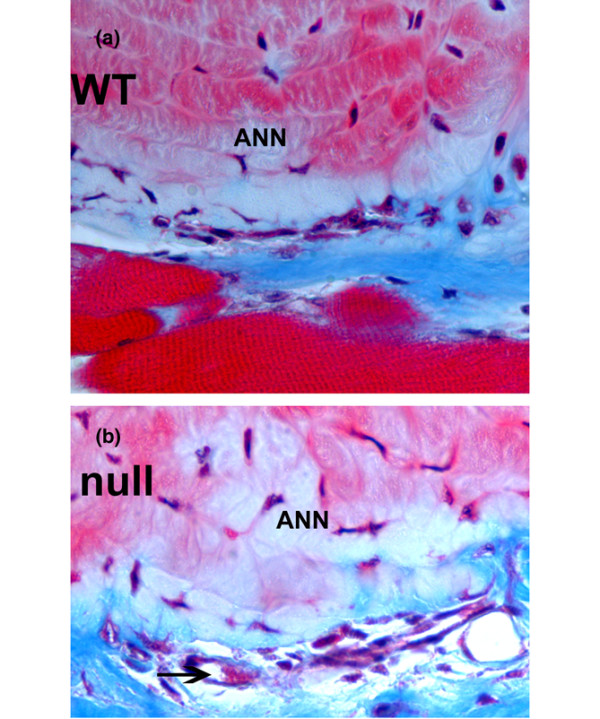
Blood vessels in soft tissues on the margin of the ventral annulus. **(a) **Wild-type (WT) specimen with modest vascularization. **(b) **Larger number of vessels (arrow) in the margin of a disc from a thrombospondin-2-null animal. Ann, annulus; arrow, capillary containing red blood cells. Masson-trichrome stain; magnification, ×600.

Vascularization in soft tissue bordering the ventral annulus in discs from WT animals was sparse (Figure 6a). Although small blood vessels containing red blood cells could be visualized in Masson-trichrome-stained tissue (Figure 6b), empty or very small vessels were difficult to identify. To overcome this problem, we performed immunolocalization of PECAM-1, which is expressed on the plasma membrane of endothelial cells [22]. PECAM-1 has been shown to recognize only vascular endothelial cells [23].

**Figure 7:**
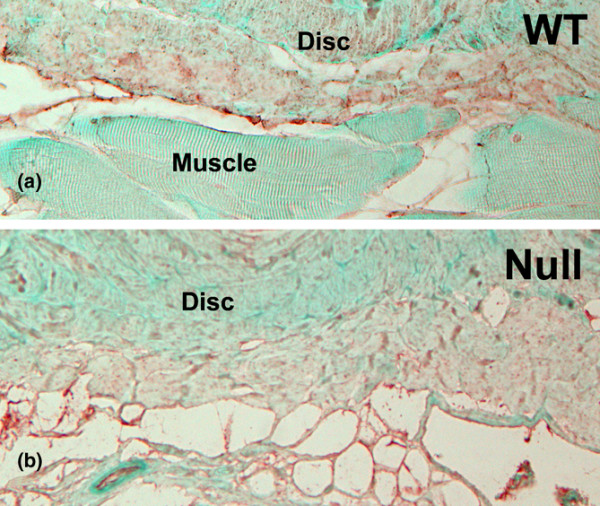
Location of small blood vessels along the margin of the ventral portion of the annulus. Small blood vessels were visualized with platelet endothelial cell adhesion molecule-1 immunohistochemistry (red localization product). **(a) **Modest vasculature is present in the tissues near the disc in the wild-type (WT) mouse. **(b) **In the thrombospondin-2-null mouse, a much larger vascular bed is present. Magnification, ×500.

Figure 7a shows a representative immunolocalization image of PECAM-1-positive vessels along the margin of discs from WT mice or TSP-2-null mice (Figure 7b). Counts of PECAM-1-positive blood vessels in tissue along the margin of the ventral annulus were performed on the margins of 20 control mouse discs and 15 TSP-2-null mouse discs. The resulting data are indicative of a significantly larger vascular bed in TSP-2-null mice compared with WT mice (19.8 ± 2.75 versus 6.2 ± 1.08, respectively; *P *= 0.0001). In addition to increased vascular numbers, the organization of these vascular beds was notably less regular in the TSP-2-null mice (Figure 7b) compared with WT control mice (Figure 7a). No vascularization within the annulus or nucleus was detected by anti-PECAM-1 IgG in either TSP-2-null mouse discs or WT mouse discs.

## Discussion

### TSP-2 in the disc

TSP-2, a matricellular protein with recognized anti-angiogenic activity *in vivo *and *in vitro*, was found to be present in some, but not all, cells of the human and mouse annulus. Microarray analysis also verified expression of TSP-2 in the human annulus.

It is interesting that the response to a lack of TSP-2 expression in null mice was insufficient to produce vascular ingrowth into the disc. The disc may indeed be unusual in this regard, since surrounding soft tissues bordering the disc displayed the expected increased vascular bed previously seen in soft tissues of the TSP-2-null animal [4]. The annulus contains TSP-2, but apparently does not rely solely upon it to inhibit blood vessel growth; TSP-2 may therefore not participate in regression of neonatal vessels in the disc.

### Morphology of the annulus in the TSP-2-null mouse

Although there was no difference in the number of disc cells in the annulus of TSP-2-null mice versus WT animals, an important finding in the present study is the irregular, uneven collagen lamellar structure seen by polarized light microscopy in null mouse discs versus WT mouse discs. Appropriate annular morphology and integrity are essential to the function of the intervertebral disc, and cells in the outer annulus are polarized for directed secretion of ECM components [24]. Ultimately it is the ECM that undergoes failure with disc degeneration; dehydration and matrix fraying culminate in tears within the annulus during biomechanical loading and torsion. Nucleus pulposus and annulus material rupture through these tears, and impinge on nerves, thus causing pain.

Kyriakides and colleagues found that dermal collagen fibers were disorganized in the TSP-2-null mouse, and that the skin of these mice displayed decreased tensile strength [4]. These authors hypothesized that TSP-2 might function as a collagen fibril-associated protein that participates in the regulation of collagen fibril diameter and fibrillogenesis. This hypothesis suggests another avenue to be explored in disc cell biology. Other possibilities include the ability of TSP-2 to regulate the activity of matrix metalloproteinase-2 [5] and tissue transglutaminase [2]. Additional studies of the disc characteristics of the TSP-2-null mouse are needed to determine whether there are ultrastructural changes in collagen fibers similar to those seen in the tendons of TSP-2-null mice (that is, increased numbers of large-diameter fibrils) and to determine whether there is decreased tensile strength in TSP-2-null discs.

### Vascular changes in soft tissue bordering the disc in the TSP-2-null mouse

Although there is a rich vascular supply to the developing and newborn disc, vascularity decreases with maturity, and the adult human disc is avascular. This condition is also present in many other species, including the sand rat, a small rodent model of spontaneous, age-related disc degeneration [25,26].

The small vascular beds along the dorsal and ventral annular surfaces, and vascularization of the vertebral endplate, constitute the main accesses to vasculature for the disc, and nutrients subsequently reach the cells of the disc via diffusion through the disc ECM. In humans and in some animals, including the sand rat, the endplate undergoes calcification with increasing age, and access to nutrients thus decreases further [26-31].

Previous work has shown that the majority of cells in the outer annulus of the human disc and the sand rat disc contain TSP-1. Since TSP-2 has established anti-angiogenic properties, we hypothesized that this matricellular protein might contribute to the avascular state of the disc by its capacity to inhibit vascular ingrowth along the disc margin. One objective of this work was to examine mice with a targeted disruption of the TSP-2 gene to determine whether mice lacking TSP-2 would show enhanced vascularity of the adult annulus. Our studies show that, even in the absence of expression of the TSP-2 gene, vascular ingrowth into the body of the disc did not occur.

The results of the quantitative assessment of the vascular bed in soft tissue along the disc margins that we presented here are similar to those previously published by Kyriakides and colleagues, who counted blood vessels in adipose, dermis, and thymic tissues of WT mice and TSP-2-null mice [4]. A similar increase in vasculature was therefore seen in the TSP-2-null mouse tissue in the margin of the disc. It is interesting to note that the previous investigators also showed that the differences seen in neonatal or embryonic dermis were not as great as those in adult tissue.

In the TSP-2-null specimens examined here, the expected increased vascular bed was present in the soft tissue margin adjacent to the disc. Although we have not measured any nutrient diffusion rates in these discs, we note that this increased adjacent vascularity may potentially result in an increased availability of nutrients to the disc.

It is also of interest to comment on the question of potential compensation between the two TSPs. There is no evidence to date for compensation. Although we have not tested whether upregulation of TSP1 might contribute to the lack of vascularization of the annulus in TSP-2-null mice, the evidence in the literature suggests that compensation by either of the two TSPs does not occur [32].

Angiogenesis is seen in herniated disc tissue, and basic fibroblast growth factor has been noted in at least some of the blood vessels in the prolapsed disc. In contrast, no immunoreactivity for fibroblast growth factor was seen in intact, nonherniated discs [33]. Vascular endothelial growth factor and platelet-derived growth factor have also been identified in herniated disc tissue [34-37].

There is a complex biological relationship between the intact disc matrix and cells, and the nearby vasculature outside the disc. We now recognize a number of inhibitors of angiogenesis, as discussed in several reviews [38-42]. There are angiogenesis inhibitors that function by inhibition of one, or more than one, angiogenic protein. Endogenous anti-angiogenic proteins have a number of interesting properties [43]; some can specifically target newly-formed vasculature, but not older blood vessels. Relevant to the disc, tissue inhibitor of metalloproteinase-1 and ECM fragments, including those from collagen, merit further study of their anti-angiogenic potential. Also relevant to the disc are the findings that aggrecan may act in an anti-angiogenic factor [44], as may other matrix proteoglycan components [45].

Chondromodulin-I, which is present in the disc [46], also acts as an endothelial cell growth inhibitor in fetal bovine cartilage, in growth plates, and in embryonic cartilaginous sites. In addition to TSPs, this matrix protein might exert an anti-angiogenic influence in the TSP-2-null mouse discs studied here. The work presented here points to the importance of additional studies of TSP-1-null mice and TSP-2-null mice. In addition, future studies of anti-angiogenic factors in the disc are needed to understand the change from the well-vascularized status of the fetal and young discs to the avascular adult human disc or small mammalian disc.

## Conclusion

There is a complex biological relationship between the intact disc matrix and cells and the nearby vasculature outside the disc. We have shown that although an increase in vasculature was present in the TSP-2-null tissue in the margin of the disc, vascular ingrowth into the body of the disc did not occur. The present study also identified a change in lamellar collagen structure in the annulus of the TSP-2-null mouse disc. Our results point to the need for future research to understand the transition from the well-vascularized status of the fetal and young discs to the avascular state of the adult human or small mammalian disc.

## Abbreviations

ECM = extracellular matrix; PECAM = platelet endothelial cell adhesion molecule-1; TSP = thrombospondin; WT = wild type.

## Competing interests

The authors declare that they have no competing interests.

## Authors' contributions

HEG, PB and EHS participated in the design of the study, secured funding, contributed to the design and coordination of the study, and participated in data interpretation and extensive preparation and revision of the manuscript. JAI and NZ performed histologic studies and assisted with manuscript preparation. HJN assisted with statistical analyses. ENH Jr assisted with study design and data analysis. All authors read and approved the final manuscript.

## References

[B1] Dejong V, Degeorges A, Filleur S, Ait-Si-Ali S, Mettouchi A, Bornstein P, Binétruy B, Cabon F (1999). The Wilms' tumor gene product represses the transcription of thrombospondin 1 in response to overexpression of c-Jun. Oncogene.

[B2] Agah A, Kyriakides TR, Bornstein P (2005). Proteolysis of cell-surface tissue transglutaminase by matrix metalloproteinase-2 contributes to the adhesive defect and matrix abnormalities in thrombospondin-2-null fibroblasts and mice. Am J Pathol.

[B3] Bornstein P, Armstrong LC, Hankenson KD, Kyriakides TR, Yang Z (2000). Thrombospondin 2, a matricellular protein with diverse functions. Matrix Biol.

[B4] Kyriakides TR, Zhu Y-H, Smith LT, Bain SD, Yang Z, Lin MT, Danielson KG, Iozzo RV, LaMarca M, McKinney CE, Ginns EI, Bornstein P (1998). Mice that lack thrombospondin 2 display connective tissue abnormalities that are associated with disordered collagen fibrillogenesis, an increased vascular density, and a bleeding diathesis. J Cell Biol.

[B5] Yang Z, Kyriakides TR, Bornstein P (2000). Matricellular proteins as modulators of cell-matrix interactions: adhesive defect in thrombospondin 2-null fibroblasts is a consequence of increased levels of matrix metalloproteinase-2. Mol Biol Cell.

[B6] Simantov R, Febbraio M, Silverstein RL (2005). The antiangiogenic effect of thrombospondin-2 is mediated by CD36 and modulated by histidine-rich glycoprotein. Matrix Biol.

[B7] Breitkopf K, Sawitza I, Westhoff JH, Wickert L, Dooley S, Gressner AM (2005). Thrombospondin 1 acts as a strong promoter of transforming growth factor β effects via two distinct mechanisms in hepatic stellate cells. Gut.

[B8] Schultz-Cherry S, Chen H, Mosher DF, Misenheimer TM, Krutzsch HC, Roberts DD, Murphy-Ulrich JE (1995). Regulation of transforming growth factor-beta activation by discrete sequences of thrombospondin 1. J Biol Chem.

[B9] Hankenson KD, Bain SD, Kyriakides TR, Smith EA, Goldstein SA, Bornstein P (2000). Increased marrow-derived osteoprogenitor cells and endosteal bone formation in mice lacking thrombospondin 2. J Bone Mineral Res.

[B10] Hankenson KD, Ausk BJ, Bain SD, Bornstein P, Gross TS, Srinivasan S (2006). Mice lacking thrombospondin 2 show an atypical pattern of endocortical and periosteal bone formation in response to mechanical loading. Bone.

[B11] Gruber HE, Ingram JA, Hanley EN (2006). Immunolocalization of thrombospondin in the human and sand rat intervertebral disc. Spine.

[B12] Crock HV, Goldwasser M, Yoshizawa H, Ghosh P (1988). Vascular anatomy related to the intervertebral disc. The Biology of the Intervertebral Disc.

[B13] Sahlman J, Inkinen R, Hirvonen T, Lammi MJ, Lammi PE, Nieminen J, Lapvetelainen T, Prockop DJ, Arita M, Li SW, Hyttinen MM, Helminen HJ, Puustjarvi K (2001). Premature vertebral endplate ossification and mild disc degeneration in mice after inactivation of one allele belonging to the Col2a1 gene for type II collagen. Spine.

[B14] Li X, Leo BM, Beck G, Balian G, Anderson DG (2004). Collagen and proteoglycan abnormalities in the GDF-5-deficient mice and molecular changes when treating disk cells with recombinant growth factor. Spine.

[B15] Mátés L, Nicolae C, Mörgelin M, Deák F, Kiss I, Aszódi A (2004). Mice lacking the extracellular matrix adaptor protein matrilin-2 develop without obvious abnormalities. Matrix Biol.

[B16] O'Connell GDVEJ, Elliott DM (2007). Comparison of animals used in disc research to human lumbar disc geometry. Spine.

[B17] Elliott DM, Sarver JJ (2004). Young investigator award winner: validation of the mouse and rat disc as mechanical models of the human lumbar disc. Spine.

[B18] Thompson JP, Pearce RH, Schechter MT, Adams ME, Tsang IKY, Bishop PB (1990). Preliminary evaluation of a scheme for grading the gross morphology of the human intervertebral disc. Spine.

[B19] Gruber HE, Mougeot J-L, Hoelscher GL, Ingram JA, Hanley EN (2007). Microarray analysis of laser capture microdissected annulus cells from the human intervertebral disc. Spine.

[B20] Kyriakides TR, Zhu Y-H, Yang Z, Bornstein P (1998). The distribution of the matricellular protein thrombospondin 2 in tissues of embryonic and adult mice. J Histochem Cytochem.

[B21] Ramirez MI, Pollack K, Millien G, Cao YX, Hinds A, Williams MC (2002). The alpha-isoform of caveolin-1 is a marker of vasculogenesis in early lung development. J Histochem Cytochem.

[B22] Scholz D, Schaper J (1997). Platelet/endothelial cell adhesion molecule-1 (PECAM-1) is localized over the entire plasma membrane of endothelial cells. Cell Tissue Res.

[B23] Ricono JM, Xu Y-C, Arar M, Jin D-C, Barnes JL, Abboud HE (2003). Morphological insights into the origin of glomerular endothelial and mesangial cells and their precursors. J Histochem Cytochem.

[B24] Gruber HE, Ingram J, Hoelscher GL, Norton HJ, Hanley EN (2007). Cell polarity in the anulus of the human intervertebral disc. Morphologic, immunocytochemical, and molecular evidence. Spine.

[B25] Gruber HE, Johnson T, Norton HJ, Hanley EN (2002). The sand rat model for disc degeneration: Radiologic characterization of age-related changes. Cross-sectional and prospective analyses. Spine.

[B26] Gruber HE, Ashraf N, Kilburn J, Williams C, Norton HJ, Gordon BE, Hanley EN (2005). Vertebral endplate architecture and vascularization: application of micro-computerized tomography, a vascular tracer, and immunocytochemistry in analyses of disc degeneration in the aging sand rat. Spine.

[B27] Modic MT, Steinberg PM, Ross JS, Masaryk TJ, Carter JR (1988). Degenerative disk disease: assessment of changes in vertebral body marrow with MR imaging. Radiology.

[B28] Horner HA, Urban JPG (2001). 2001 Volvo Award winner in basic science studies: effect of nutrient supply on the viability of cells from the nucleus pulposus of the intervertebral disc. Spine.

[B29] Holm SH, Weinstein JN, Wiesel SW (1994). Nutrition of the intervertebral disc. The Lumbar Spine.

[B30] Maroudas A, Ghosh P (1988). Nutrition and metabolism of the intervertebral disc. The Biology of the Intervertebral Disc.

[B31] Maroudas A, Stockwell RA, Nachemson A, Urban J (1975). Factors involved in the nutrition of the human lumbar intervertebral disc: cellularity and diffusion of glucose *in vitro*. J Anat.

[B32] Agah A, Kyriakides TR, Lawler J, Bornstein P (2002). The lack of thrombospondin-1 (TSP1) dictates the course of wound healing in double-TSP1/TSP2-null mice. Am J Pathol.

[B33] Tolonen J, Gronblad M, Virri J, Seitsalo S, Rytomaa T, Karaharju E (1995). Basic fibroblast growth factor immunoreactivity in blood vessels and cells of disc herniations. Spine.

[B34] Tolonen J, Grönblad M, Virri J, Seitsalo S, Rytömaa T, Karaharju EO (1997). Platelet-derived growth factor and vascular endothelial growth factor expression in disc herniation tissue: an immunohistochemical study. Eur Spine J.

[B35] Kato T, Haro HKH, Shinomiya K (2004). Sequential dynamics of inflammatory cytokine, angiogenesis inducing factor and matrix degrading enzymes during spontaneous resorption of the herniated disc. J Orthop Res.

[B36] Koike Y, Uzuki M, Kokubun S, Sawai T (2003). Angiogenesis and inflammatory cell infiltration in lumbar disc herniation. Spine.

[B37] Haro H, Kato T, Komori H, Osada M, Shinomiya K (2002). Vascular endothelial growth factor (VEGF)-induced angiogenesis in herniated disc resorption. J Orthop Res.

[B38] Nyberg P, Xie L, Kalluri R (2005). Endogenous inhibitors of angiogenesis. Cancer Res.

[B39] Sottile J (2004). Regulation of angiogenesis by extracellular matrix. Biochem Biophys Res Commun.

[B40] Folkman J (2004). Endogenous angiogenesis inhibitors. APMIS.

[B41] Folkman J (2006). Antiangiogenesis in cancer therapy – endostatin and its mechanisms of action. Exp Cell Res.

[B42] Nakamura T, Matsumoto K (2005). Angiogenesis inhibitors: from laboratory to clinical application. Biochem Biophys Res Commun.

[B43] Cao Y (2004). Antiangiogenic cancer therapy. Semin Cancer Biol.

[B44] Johnson WEB, Caterson B, Eisenstein SM, Roberts S (2005). Human intervertebral disc aggrecan inhibits endothelial cell adhesion and cell migration *in vitro*. Spine.

[B45] Melrose J, Roberts S, Smith S, Menage J, Ghosh P (2002). Increased nerve and blood vessel ingrowth associated with proteoglycan depletion in an ovine anular lesion model of experimental disc degeneration. Spine.

[B46] Takao T, Iwaki T, Kondo J, Hiraki Y (2000). Immunohistochemistry of chondromodulin-I in the human intervertebral discs with special reference to the degenerative changes. Histochem J.

